# Concentration of anti-pneumococcal capsular polysaccharide IgM, IgG and IgA specific antibodies in adult blood donors

**DOI:** 10.1016/j.plabm.2016.02.004

**Published:** 2016-02-24

**Authors:** Antony R. Parker, Syreeta Allen, Stephen Harding

**Affiliations:** The Binding Site Group Limited, 8 Calthorpe Rd, Birmingham B15 1QT, UK

**Keywords:** Anti-pneumococcal capsular polysaccharide IgG, Anti-pneumococcal capsular polysaccharide IgA, Anti-pneumococcal capsular polysaccharide IgM, Pneumovax, Antibody deficiency, Polysaccharide

## Abstract

**Objectives:**

Anti-pneumococcal capsular polysaccharide (PCP) IgM, IgG and IgA ELISAs have been developed to aid assessment of the adaptive immune system. The relationship between the concentrations of PCP IgM, IgG, and IgA was investigated.

**Design and methods:**

The concentrations of PCP IgM, IgG, and IgA were measured in sera obtained from 231 adult blood donors.

**Results:**

Concentrations of each isotype were not normally distributed. The median concentration for PCP IgM was 54 U/mL (range 37–75 U/mL), IgG 40 mg/L (range 26–79 mg/L) and IgA 21 U/mL (range 13–44 U/mL). The median PCP IgM titres decreased with age and were significantly lower in patients aged 81–90 years compared to those aged 18–80 years. By contrast, there was a significantly higher median serum PCP IgG titre in the 61–90 years group compared to those aged 18–60 years and a significantly higher median serum PCP IgA titre in the 51–90 years group compared to those aged 18–50 years. The correlation between PCP IgG and IgA was more significant than between IgM and IgA and between IgM and IgG. Correlation of PCP IgA and IgM concentrations identified four phenotypes: high PCP IgM and IgA; high PCP IgM only; high PCP IgA only; and low PCP IgM and IgA. A significant number of individuals with a PCP IgG concentration >50 mg/L had low PCP IgA and IgM concentrations.

**Conclusion:**

The additional measurement of PCP IgA and PCP IgM, alongside PCP IgG, in individuals investigated for a compromised immune system may provide a more detailed antibody profile.

## 1 Introduction

Serum antibody measurements are used to assess immune system competence and recovery, and are included in guidelines for the assessment of antibody deficiencies [1], [5], [7]. Commonly measured antibodies include those raised in response to tetanus, haemophilus and pneumococcal capsular polysaccharide (PCP). Recently, the measurement of PCP IgM and IgA has been reported in patients with common variable immunodeficiency (CVID) [3]. Cavaliere et al. identified four PCP IgM and IgA phenotypes and assessed their concentrations retrospectively to stratify the risk of pneumonia and bronchiectasis. At present, the measurement of antigen-specific IgA and IgM is not routinely performed for the assessment of immunocompetence or risk of infection.

In this study we report the concentration of, and correlation between, PCP IgM, IgG, and IgA antibodies in a large cohort of blood donor samples. We hypothesise that the simultaneous measurement IgA and IgM in addition to PCP IgG may give the clinician a more detailed antibody profile for the assessment of immunocompetence.

## 2 Materials and methods

### 2.1 Samples

Serum samples were obtained from 231 blood donors (125 males and 106 females) aged 18–90 years. Only subjects who were free of recurrent infections or inflammation at the time of donation (assessed by questionnaire) and whose C-reactive protein concentrations were <10 mg/L were included in the analysis. Samples were stored at −80 °C. The samples were collected in donor centres by Biomex Solutions (Heidelberg, Germany) and purchased from Quest Biomedical (Solihull, UK). Sample collection was approved by the Institution Ethics Review Board (#05142), with all donors providing written informed consent.

### 2.2 Measurement of antigen-specific antibodies

Commercially available test kits (VaccZyme™ PCP Enzyme-Linked Immunosorbent Assays [ELISAs], The Binding Site Group Limited, Birmingham, UK) were used to measure PCP IgM, IgG and IgA according to the manufacturer’s instructions. All three ELISAs employ the use of capsular polysaccharide (CPS) absorption to improve the specificity of pneumococcal antibody detection. The data obtained was stratified by age (Table 1) and median values and interquartile ranges (IQR) were established for each age group. Median values were used to establish cut-offs for anti-PCP IgA and IgM, which were compared to previously published cut-offs (20 U/mL and 150 U/mL respectively) for defining four phenotypic groups [3]. For PCP IgG, the cut-off concentration of 50 mg/L was applied [4].

**Table 1:** Age stratified median PCP IgM, IgG and IgA antibody titres and interquartile ranges.

	Age stratified median antibody titre (interquartile range)							
	**18–20 yrs**	**21–30 yrs**	**31–40 yrs**	**41–50 yrs**	**51–60 yrs**	**61–70 yrs**	**71–80 yrs**	**81–90 yrs**
**PCP IgM (U/mL)**	65	57	54	46	56	50	44	27
	(47–156)	(45–81)	(36–94)	(32–72)	(34–73)^a^	(29–77)	(28–83)^a^	(14–52)^a^
**Number of samples**	12	78	18	37	25	29	21	11
**PCP IgG (mg/L)**	32	32	34	46	37	98	110	67
	(17–49)^a^	(22–54)	(30–49)	(27–76)	(26–69)	(28–168)	(58–270)	(48–117)
**Number of samples**	12	78	18	37	23	28	21	11
**PCP IgA (U/mL)**	20	19	21	24	28	35	43	55
	(11–27)^a^	(12–29)	(12–27)	(13–51)	(15–67)	(14–116)	(43–96)	(21–96)^a^
**Number of samples**	13	77	18	36	25	29	21	11

a These samples were determined to be normally distributed by Shapiro Wilk analysis.

### 2.3 Statistical analysis

Shapiro–Wilk, Chi squared, Fisher’s exact and Mann Witney *U* tests were performed using Graphpad Prism software (GraphPad Software, La Jolla, CA, USA). *p*<0.05 was considered statistically significant.

## 3 Results

The Shapiro–Wilk test for Gaussian distribution demonstrated that PCP IgM, IgG and IgA concentrations from adult blood donors were not normally distributed (*p*<0.0001). The median adult concentrations (18–90 years) and IQR ranges (25–75%) were as follows: PCP IgM: median 54 U/ml, range 37–75 U/ml (*n*=231); PCP IgG: median 40 mg/L, range 26–79 mg/L (*n*=228); and PCP IgA: median 21 U/ml, range 13–44 U/ml (*n*=230).

**Fig. 1:**
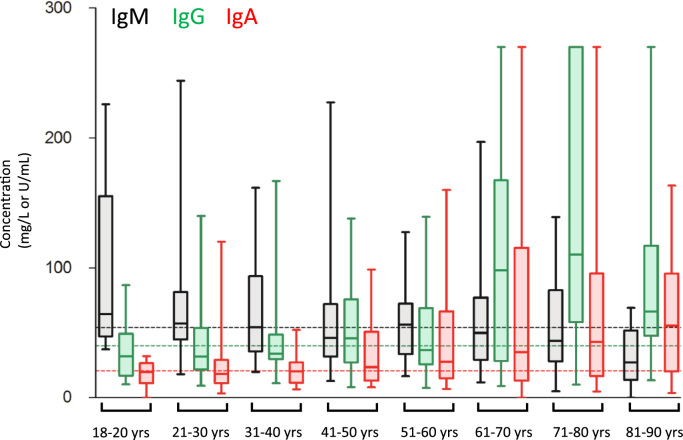
Age-stratified concentrations of PCP IgM (U/mL), IgG (mg/L) and IgA (U/mL) in adult blood donors. Dotted lines represent median concentrations for IgM (54 U/mL; black), IgG (40 mg/L; green) and IgA (21 U/mL; red). Box and Whisker plots show interquartile ranges, median, minimum and maximum concentrations.

Fig. 1 shows the age-stratified adult PCP IgM, IgG and IgA concentrations in normal blood donors. 51% (115/226) of samples had a PCP IgM concentration greater than the median (54 U/mL). The median PCP IgM titres decreased with age and there was a significantly lower median PCP IgM titre in patients aged 81–90 years (27 U/mL) compared to those aged 18–80 years (55 U/mL; *p*=0.0017). 50% (114/226) of samples had a PCP IgG concentration greater than the median (40 mg/L). There was a significantly higher median PCP IgG titre in the 61–70 years (98 mg/L; *p*=0.0004), 71–80 years (110 mg/L; *p*<0.0001), and 81–90 years (67 mg/L; *p*=0.018) groups compared to those aged 18–60 years (median 35 mg/L). For PCP IgA, 50% (113/226) of samples had a concentration greater than the median (21 U/mL). There was a significantly higher median PCP IgA titre in the 51–60 years (28 U/mL; *p*=0.036), 61–70 years (35 U/mL; *p*=0.0078), 71–80 years (43 U/mL; *p*=0.0065), and 81–90 years (55 U/mL; *p*=0.0034) groups compared to those aged 18–50 years (median 20 U/mL). The age-specific concentration ranges for PCP IgM, IgG and IgA are shown in Table 1.

The correlation between PCP IgG and IgA was more significant (*r*=0.45, *p*<0.0001) than the correlation between IgM and IgA (*r*=0.2, *p*=0.003), or between IgM and IgG (*r*=0.012, *p*=0.86) (Table 2). The number of samples with concentrations above and below the medians was highly correlated between PCP IgG and IgA (*p*<0.0001). When a concentration cut-off of 50 mg/L was used for PCP IgG, PCP IgG and IgA were again highly correlated (*p*<0.0001) (Table 3). When published cut-offs were used for PCP IgM and PCP IgA levels (20 U/mL and 150 U/mL, respectively), the correlation between PCP IgG and IgA was higher than the correlation between the other pairs.

**Table 2:** Correlation between PCP IgM, IgG and IgA concentrations. Values top right of the grey boxes are from linear regression analysis. Values bottom left of the grey boxes are from the Fisher's exact test using median concentrations as cut-offs.

	**Linear regression analysis**			
**Samples above or below median concentrations**		PCP IgM	PCP IgG	PCP IgA
	PCP IgM		*r*=0.012 *p*=0.86	*r*=0.20 *p*=0.003
	PCP IgG	*p*=0.79		*r*=0.45 *p*<0.0001
	PCP IgA	*p*=0.69	*p*<0.0001

**Table 3:** Correlation between PCP IgM, IgG and IgA concentrations according to published cut-offs and median values. Values top right of the grey boxes are produced using the following cutoffs: PCP IgM=20 U/mL, PCP IgG=50 mg/L and PCP IgA=150 U/mL [3], [4]. Values bottom left of the grey boxes are produced using the median values for PCP IgM and IgA (54 U/mL and 21 U/mL, respectively) and 50 mg/L for PCP IgG. All values were obtained using the Fisher's exact test.

	**Samples above or below published PCP IgM, IgG and IgA cut-offs**[3], [4]			
**Samples above or below median concentrations (for PCP IgM and IgA) or 50 mg/L (for PCP IgG)**		PCP IgM	PCP IgG	PCP IgA
	PCP IgM		*P*=0.79	*P*=1.00
	PCP IgG	*P*=0.69		*P*=0.07
	PCP IgA	*P*=0.69	*P*<0.0001	

**Fig. 2:**
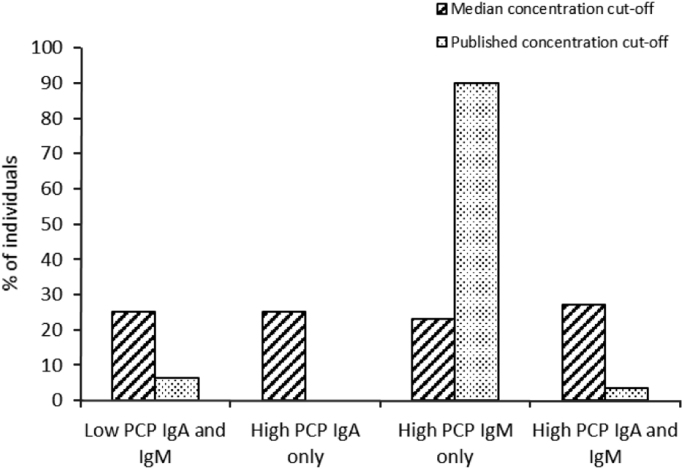
Distribution of PCP IgM and IgA phenotypes according to median or published concentration cut-offs (*n*=226). Median concentration cut-offs were 54 U/ml and 21 U/mL for PCP IgM and PCP IgA, respectively. Published concentration cut-offs were 20 U/mL and 150 U/mL for PCP IgM and PCP IgA, respectively [3].

Four phenotypes could be differentiated using median concentrations of PCP IgA and IgM (Fig. 2): (i) high PCP IgM and IgA, (ii) high PCP IgM only, (iii) high PCP IgA only and (iv) low PCP IgM and IgA. The percentage of individuals with each phenotype was similar. Separation of the samples by PCP IgA and IgM concentrations of 150 U/mL and 20 U/mL respectively [3] significantly changed the number of individuals within each phenotypic group (high PCP IgM only>Low PCP IgM and IgA>High PCP IgM and IgA>High PCP IgA only).

**Fig. 3:**
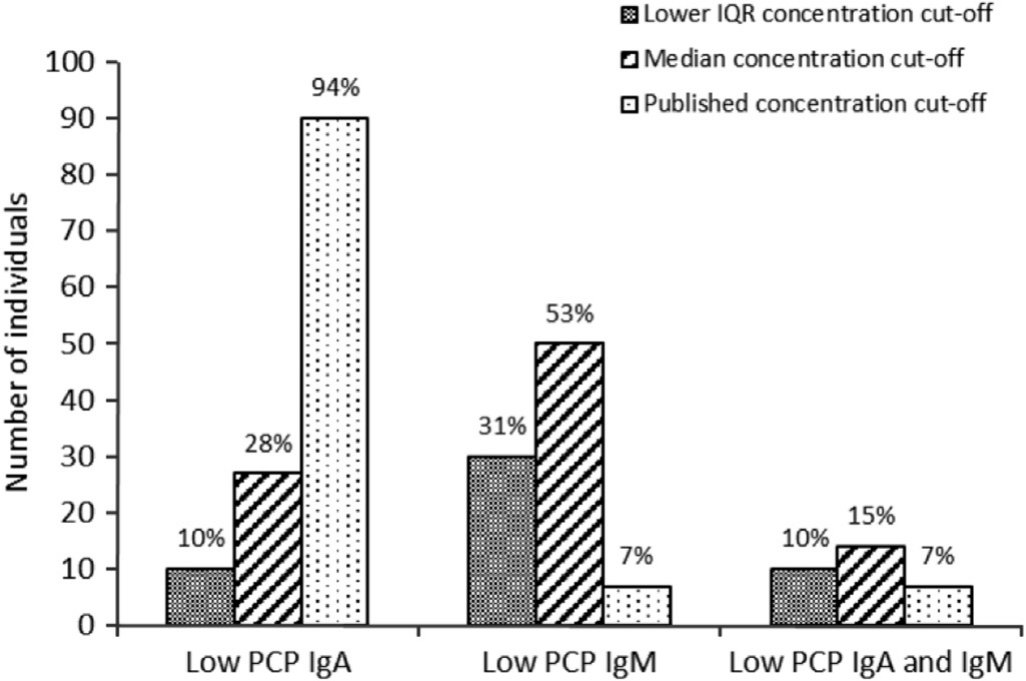
Numbers and percentages of individuals with PCP IgG concentration>50 mg/L (*n*=97) but low PCP IgM and IgA concentrations. 25th centile (lower IQR) concentration cut-offs were 37 U/mL and 13 U/mL for PCP IgM and PCP IgA, respectively. Median concentration cut-offs were 54 U/mL and 21 U/mL for PCP IgM and PCP IgA, respectively. Published concentration cut-offs were 20 U/mL and 150 U/mL for PCP IgM and PCP IgA, respectively [3].

PCP IgA and IgM concentrations were measured in samples with a PCP IgG concentration>50 mg/L (Fig. 3). 10–94% had PCP IgA lower, 7–53% had PCP IgM lower and 7–15% of samples had a concentration of both PCP IgA and IgM lower than the 25th centile (lower IQR) of the normal range, the median values or the concentration cut offs previously reported [3].

## 4 Discussion

Measurement of PCP antibody concentrations currently relies on the IgG isotype only whereas assessment should be multi-isotypic to enable complete identification of a deficient antibody response. The purpose of this study was to determine the isotype-specific antibody titres in age defined populations to provide a baseline for subsequent studies.

The production of PCP IgM is pivotal for the protection against pneumococcal disease [2], [3], [6] and has been shown to be defective in CVID [3]. CVID patients with PCP IgA and IgM concentrations above 150 U/mL and 20 U/mL, respectively have been shown to have a lower risk of developing pneumonia and bronchiectasis than those with either a PCP Ig Mconcentration<20 U/mL only or those with both concentrations below both the above cut-offs [3]. In this study, using the same cut-offs in adult blood donors, the majority of individuals had a PCP IgM concentration>20 U/mL. Schutz and colleagues [8] have previously reported an IgA and IgM response in all healthy individuals vaccinated with Pneumovax (8/10 PCP IgA>150 U/mL, with the 2/10<150 U/mL possessing a >5 fold increase in concentration post-vaccination, and 10/10 PCP IgM>20 U/mL). Cavaliere reported that the pre-vaccination PCP IgM concentration in CVID patients was 10.2±24.9 U/mL, and for PCP IgA 11.6±29.1 U/mL. Using the baseline levels from this study a number of CVID patients may have both IgM and IgA antibody levels lower that the base line concentration in the normal blood donor population.

The median concentration of PCP IgM antibodies decreased with age. The opposite was true of PCP IgG and IgA concentrations which were both stable up until approximately 60 years and then increased significantly from 61 to 90 years. A significant proportion of this older population are likely to have received a pneumococcal vaccination. Pneumovax™, which was approved by the US Food and Drug Administration (FDA) in 1983, is recommended for US individuals aged 50 years. Prevnar 7 and Prevnar 13 have been approved by the FDA more recently (in 2001 and 2010, respectively). Prior vaccination or exposure to pneumococcal pathogens in this older population may be responsible for the elevated IgA and IgG titres.

By applying median concentrations of PCP IgM and PCP IgA as cut-off values, we differentiated 4 phenotypes, with approximately equal distribution of individuals across each group (Fig. 2). Importantly, in 25% of individuals both PCP IgM and PCP IgA concentrations were lower than the median values.

The measurement of PCP IgG is used to aid the diagnosis of an antibody deficiency and can be used to support the selection of therapy or treatment in those individuals. In our study, a large percentage of adult blood donors with PCP IgG>50 mg/L possessed either low PCP IgM (7–53%) or both low PCP IgM and low PCP IgA (7–15%). A PCP IgG concentration>50 mg/L [4] did not always predict PCP IgM and IgA concentrations above the median values or published cut-offs. The measurement of PCP IgM and IgA concentrations, in this population of individuals with PCP IgG>50 mg/L, would have further identified between 7 and 14 individuals with a higher risk of pneumococcal infection. Measurement of PCP IgM and IgA may have particular utility for individuals with suspected antibody deficiency or those suffering from recurrent infections, who present with PCP IgG levels greater than 50 mg/L.

In conclusion, we report reference ranges in adult blood donors for PCP IgM, IgG and IgA concentrations, and offer an explanation for the trends observed. A substantial number of individuals in our population did not have PCP IgM, IgG or IgA concentrations suggestive of protection from infection [2], [3], [4], [6], [9]. In particular, a number of individuals had PCP IgG>50 mg/L but low levels of PCP IgM and IgA. The addition of PCP IgM and PCP IgA to the panel of tests measured in individuals investigated for a compromised immune system warrants further investigation.
